# Combined Intracerebroventricular Enzyme Replacement and Cord Blood Transplantation in Patients with Mucopolysaccharidosis Type II Diagnosed Through Newborn Screening

**DOI:** 10.3390/ijns12030047

**Published:** 2026-06-26

**Authors:** Yuki Ueda, Shinsuke Hirabayashi, Masayuki Miura, Satoshi Yamada, Sachiko Nakakubo, Midori Nakajima, Takeru Goto, Jutaro Abe, Yukayo Terashita, Atsushi Manabe, Torayuki Okuyama, Kiyoshi Egawa

**Affiliations:** 1 Department of Pediatrics, Hokkaido University Hospital, North 14, West 5, Kita-Ku, Sapporo 060-8648, Hokkaido, Japan; yu_ueda@med.hokudai.ac.jp (Y.U.); hirashin@huhp.hokudai.ac.jp (S.H.); nl_lesser@yahoo.co.jp (S.Y.); s-nakakubo@huhp.hokudai.ac.jp (S.N.); midori.nakajima@med.hokudai.ac.jp (M.N.); tgoto23@med.hokudai.ac.jp (T.G.); jutaro@med.hokudai.ac.jp (J.A.); ukeyon99@med.hokudai.ac.jp (Y.T.); atmanabe@med.hokudai.ac.jp (A.M.); 2 Hokkaido Pharmaceutical Association Public Health Examination Center, Hiragishi 1-jo, 8-chome, Toyohira-Ku, Sapporo 062-0931, Hokkaido, Japan; miura_masayuki@douyakken.or.jp; 3 Department of Genomic Medicine, Saitama Medical University Hospital, Morohongo 38, Moroyamamachi, Iruma-gun, Saitama 350-0451, Saitama, Japan; tora@ion.ocn.ne.jp

**Keywords:** mucopolysaccharidosis type II, intracerebroventricular administration, enzyme replacement therapy, cord blood transplantation

## Abstract

Enzyme replacement therapy (ERT) for central nervous system symptoms and newborn screening (NBS) is available in Japan for patients with mucopolysaccharidosis type II (MPS II). Of 12 suspected cases identified through the NBS program, 3 patients were diagnosed with neuronopathic MPS II (2 directly from the screened cohort and 1 affected sibling). We reviewed the clinical courses of these patients, who were treated with intracerebroventricular (ICV) ERT using idursulfase beta (Hunterase^®^) followed by umbilical cord blood transplantation (CBT). Heparan sulfate (HS) in the cerebrospinal fluid (CSF) was longitudinally measured as a therapeutic biomarker, and developmental age was evaluated. All patients achieved successful engraftment with no severe complications, except for one patient with sinusoidal obstruction syndrome. The CSF HS concentration showed a temporary increase during the ERT discontinuation period, which can be attributed to CBT, and a subsequent reduction after the resumption of ICV-ERT. The patients exhibited age-appropriate development. The pattern of change in HS indicates the importance of continuing ICV-ERT even after hematopoietic stem cell transplantation. The study results demonstrate that the combination of ICV-ERT and CBT may yield promising outcomes in patients with neuronopathic MPS II, underscoring the importance of early intervention through NBS.

## 1. Introduction

Mucopolysaccharidosis type II (MPS II), also known as Hunter syndrome, is a rare X-linked lysosomal storage disorder (LSD) that mostly affects males and is caused by deficiency of the lysosomal enzyme iduronate-2-sulfatase (I2S). This enzyme is encoded by the *IDS* gene (OMIM 309900) and plays an essential role in the degradation of glycosaminoglycans (GAGs). The main GAGs that accumulate in MPS II patients are heparan sulfate (HS) and dermatan sulfate. The resulting intra-lysosomal accumulation of GAGs leads to the progressive enlargement and dysfunction of multiple organs and tissues. Patients with MPS II gradually develop distinctive facial features such as an enlarged head circumference, temporal and frontal bossing, saddle nose, and thick lips. Representative symptoms include growth disturbances, joint contractures, valvular heart disease, hepatosplenomegaly, and psychomotor developmental delay [1], which are commonly classified according to the age of onset and the presence or absence of central nervous system (CNS) symptoms. Two-thirds of affected individuals have a neuronopathic phenotype [2].

The two principal therapeutic approaches for MPS II are enzyme replacement therapy (ERT) and hematopoietic stem cell transplantation (HSCT) [3]. ERT became available in the mid-2000s. Weekly intravenous administration of human recombinant idursulfase (Elaprase^®^) has been shown to result in the improvement of various conditions, including hepatosplenomegaly and obstructive respiratory problems, and increase the range of joint motion [4]. Intravenous ERT is effective in maintaining physical conditions and preventing life-threatening events [5]. However, idursulfase does not improve CNS symptoms due to its limited ability to cross the blood–brain barrier (BBB); thus, therapeutic intervention in the context of neuronopathic MPS II remains challenging [6,7]. Historically, HSCT was introduced first, having been used in patients with MPS II since the 1980s. Unlike for MPS type I, HSCT did not become the standard treatment for neuronopathic MPS II due to the rapid progression of CNS symptoms and high treatment-related mortality and morbidity; however, it has been performed relatively frequently in Japan, and may improve CNS symptoms in some patients [8]. This approach is based on the concept that donor-derived cells capable of producing deficient enzymes can traverse the BBB and enable enzymatic activity within the CNS [9]. With advances in transplantation technology, mortality and morbidity rates have decreased, and a high probability of successful engraftment can now be expected [10,11]. Consequently, HSCT has become an important treatment option, particularly for patients with early-stage MPS II [12].

A direct intrathecal ERT approach has been developed [13], and a clinical trial involving the intracerebroventricular (ICV) administration of idursulfase beta has shown favorable outcomes [14]. Idursulfase beta (Hunterase^®^), a once-monthly ICV-ERT agent administered via an intraventricular reservoir, was approved in Japan in 2021. Another therapeutic enzyme agent for neuronopathic MPS II, pabinafusp alpha (IZCARGO^®^), can cross the BBB via transferrin receptor-mediated transcytosis and has also recently become available in Japan [15,16]. Furthermore, although not available in all regions of Japan, newborn screening (NBS) for MPS II has been initiated as an extension of conventional NBS programs [17], creating an opportunity for early intervention in patients with MPS II. Thus, treatment strategies for MPS II have only recently undergone significant changes, and their efficacy has not yet been thoroughly evaluated. In this study, we examine the clinical courses of patients with MPS II who were diagnosed through NBS and treated with ICV-ERT followed by HSCT using umbilical cord blood.

## 2. Materials and Methods

### 2.1. Participants

Since November 2020, in Hokkaido, one of the local prefectures of Japan, the Hokkaido Early Diagnosis Network for Rare Diseases has implemented additional NBS as an optional screening test (paid for by consenting parents) for the early detection of primary immunodeficiency disorders and LSDs, including MPS II. The enzymes responsible for LSDs are measured centrally at the Hokkaido Pharmaceutical Association Public Health Center (Sapporo, Japan). Enzyme activity is measured using the 4-methylumbelliferone (4-MU) method in the first test, with tandem mass spectrometry (MS/MS) performed for confirmation. Our institution is one of the facilities to which infants are referred when a detailed examination is required. Among the patients referred to hospitals (including our facility) between November of 2020 and March of 2025, those diagnosed with MPS II were included in this study. We reviewed the results of initial diagnostic examinations (urinary GAGs and uronic acids) and confirmatory examinations (leukocyte I2S enzyme activity and the *IDS* variant), as well as the patients’ physical and psychological statuses. We also reviewed the clinical course of intravenous ERT with Elaprase^®^ (Takeda Pharmaceuticals America, Inc., Lincolnshire, IL, USA) followed by ICV-ERT with Hunterase^®^ (Clinigen K.K., Tokyo, Japan), including the age at initiation and complications of ERT. Parental consent was obtained for all patients.

### 2.2. The Clinical Course of Combined Therapy with ICV-ERT Followed by Cord Blood Transplantation

In collaboration with our facility’s pediatric hematologists, we developed a treatment strategy that combines HSCT using cord blood transplantation (CBT) and ICV-ERT. Intravenous ERT was terminated and ICV-ERT was withdrawn during the acute phase of CBT. ICV-ERT alone was resumed at one month after engraftment was achieved and the acute phase resolved.

#### 2.2.1. Indication and Protocol of CBT

Busulfan- and cyclophosphamide-based conditioning regimens were indicated. Tacrolimus and methotrexate were used to treat graft-versus-host disease (GVHD). We reviewed clinical data relating to the CBT for each patient. The following data were collected: age at indication for CBT, human leukocyte antigen (HLA) compatibility, number of CD34-positive cells/kg, period of neutrophil engraftment, platelet count > 50,000, grades of acute and chronic GVHD, and other acute and chronic adverse events. We also investigated leukocyte I2S enzyme activity after transplantation and in the interval between the resumption of ICV-ERT.

#### 2.2.2. The Longitudinal Measurement of HS in Cerebrospinal Fluid

HS in the cerebrospinal fluid (CSF) was measured every 3 months, using CSF samples collected before the administration of idursulfase beta. The GAGs were cleaved into disaccharides by methanolysis using methanolic hydrochloric acid and 2,2-dimethoxypropane, based on the internal disaccharide approach. HS concentrations were measured via liquid chromatography–tandem mass spectrometry (LC-MS/MS) at the Toray Research Center (Kamakura, Japan) [14,18,19].

#### 2.2.3. Evaluation of Developmental Age and Quotient

Developmental age (DA) and developmental quotient (DQ) were evaluated every 12 months using the Kyoto Scale of Psychological Development (KSPD) 2020, a revised (re-standardized) version of the KSPD 2001. The KSPD is widely used in Japan for developmental assessments and measures children’s developmental status in three domains: motor, cognitive–adaptive, and language–social.

## 3. Results

### 3.1. The Clinical Course of Combined Therapy of ICV-ERT with CBT

#### 3.1.1. The Profiles of Participants

Three patients were included in the study (Table 1). Between November of 2020 and March of 2025, 73,836 out of 104,514 newborns (70.6%) underwent this additional screening. Twelve infants were referred for further evaluation at hospitals, including our institution, as their I2S activity levels measured via the MS/MS method were below the cutoff value (recall rate, 0.016%). Following comprehensive diagnostic evaluation, 2 of these 12 infants (both evaluated at our institution) were diagnosed with MPS II, as described below, whereas the remaining 10 infants were determined to be false positives (positive predictive value, 16.7%).

In two patients (Patient 1 and 2), urinary GAGs were elevated and leukocyte I2S enzyme activity was absent. Urinary uronic acids were also elevated in Patient 1; however, this measurement was not performed in Patient 2. Gene testing identified a hemizygous *IDS* variant that was suspected to be pathogenic in Patient 1 (c. 685 C > T), previously reported in the severe MPS II phenotype [20], and another in Patient 2 (c. 1180 + 1 G > T), which is due to abnormal splicing. As no neurological symptoms were observed in the two infants diagnosed with NBS, considering the natural course of MPS II, intravenous ERT was initiated at 3 and 6 months of age. We prepared the intervention for the CNS by continuing weekly intravenous ERT. Regarding treatments for CNS symptoms, administration of the BBB-crossing ERT pabinafusp alfa (IZCARGO^®^) has been reported to lead to decreased CSF HS concentrations. However, in the neuronopathic type, only one patient showed an increase in DA with age; in most patients, a stagnation or decline in DA was observed [15]. In contrast, no regression was observed among patients treated with ICV-ERT using idursulfase beta (Hunterase^®^), and a sustained increase in DA was noted in multiple patients who received treatment before the age of 3 years [14]. Considering the difference in expected clinical efficacy, we provided detailed explanations to the patients’ parents and obtained their consent before deciding to administer ICV-ERT. After intraventricular reservoir implantation (Ommaya reservoir) and once-monthly ICV-ERT, idursulfase beta (Hunterase^®^) was administered at 6 and 11 months of age.

In addition, the older brother of Patient 2 was referred to our institution at the age of 2 years and 3 months (Patient 3). Because NBS had not yet been implemented at the time of his birth, I2S data from the neonatal period were unavailable. The patient had mild frontal bossing and an ectopic Mongolian spot on his back. Although he had no other representative MPS II-related symptoms, his cognitive development was slightly slower than expected for his age, and he had problems with verbal and non-verbal communication. He had high urinary GAG levels, no leukocyte I2S enzyme activity, and the same *IDS* variant as Patient 2, c. 1180 + 1G > T. As developmental delays appeared, therapeutic interventions for the CNS were quickly initiated. Following weekly intravenous ERT at the age of 2 years and 5 months, Ommaya reservoir implantation and ICV-ERT were indicated at the age of 2 years and 7 months.

#### 3.1.2. The Clinical Course of Combined Therapy

The age at CBT ranged from 1 year 8 months to 3 years 3 months (Table 2). All three patients achieved successful engraftment, ranging from days 15 to 22. The number of days until the platelet count exceeded 50,000, indicating bone marrow recovery, ranged from 32 to 62. Severe acute GVHD (grade III) was not observed. Patient 1 developed sinusoidal obstruction syndrome (SOS), a life-threatening complication. The patient responded to intensive care, including defibrotide administration, and recovered after one month. None of the patients exhibited symptoms of chronic GVHD. A late complication, hypothyroidism, occurred in patient 1. Hormonal analysis after CBT revealed a hypothyroid pattern and thyroid hormone replacement therapy was indicated. The other two cases had no apparent late complications of CBT.

In all three cases, leukocyte I2S enzyme activity increased to an adequate level, ranging from 57.9 to 132.9 nmol/mg protein/4 h. After a drug-free period of 2 to 2.5 months, ICV-ERT was resumed. Although monthly hospital visits were required for ICV-ERT, the patients were able to live their daily lives without significant restrictions.

#### 3.1.3. The Longitudinal Measurement of HS in CSF

**Figure 1:**
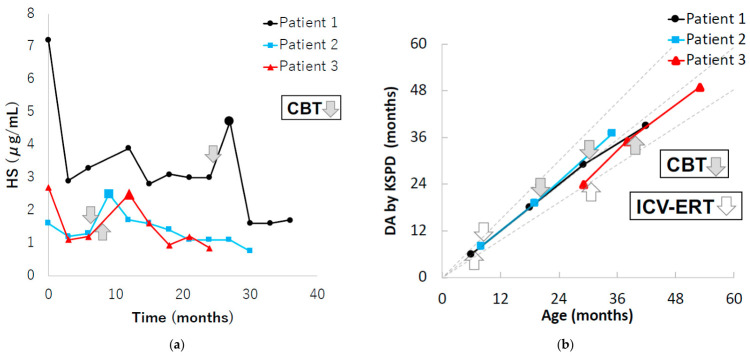
Longitudinal changes in CSF HS concentration and developmental age throughout treatment. (**a**) The longitudinal change in CSF HS concentration. The larger markers are the data before re-starting ICV-ERT due to CBT and the gray arrows indicate the timing of CBT. In all three patients, the common pattern of a temporary increase followed by a rapid decrease in HS was observed. (**b**) The change in developmental age throughout the course of treatment. The developmental age (DA) was evaluated every 12 months using the Kyoto Scale of Psychological Development (KSPD). The gray dashed lines illustrate the normal range of DA along with chronological age. The white and gray arrows represent the timing of ICV-ERT and CBT, respectively. All patients showed an increase in DA within the normal range.

In Patients 1 and 3, the HS concentration decreased immediately within the first 3 months after the start of idursulfase beta treatment. In Patient 2, the HS concentration was not elevated before treatment and remained low (Figure 1). In all three cases, the HS concentration increased during idursulfase beta withdrawal due to CBT, then decreased and remained at an acceptable level after re-administration of idursulfase beta.

#### 3.1.4. The Longitudinal Evaluation of DA and DQ

Patients 1 and 2 were diagnosed using NBS and exhibited age-appropriate DA. In Patient 3, DA was delayed by 3 months, and the total DQ score was 83 (Posture–Motor, 68; Cognitive–Adaptive, 86; Language–Social, 84). The development of Patient 3 caught up, and the DQ reached the same level as expected for his age. All three patients showed age-appropriate development (Figure 1).

## 4. Discussion

We performed ICV-ERT followed by CBT for HSCT in patients with MPS II with no or very early-stage CNS symptoms diagnosed through NBS, and observed increases in DA that paralleled chronological age. In a previous ICV-ERT study by Seo et al. [14], although an increase in DA was observed—particularly in patients whose treatment was initiated before the age of 3 years—it did not progress at a rate comparable to that of chronological age. These findings highlight the importance of initiating therapeutic interventions before irreversible damage occurs in patients with neuronopathic MPS II. Prior to our study, Ikari et al. reported favorable developmental outcomes until the age of 5 years after early intervention with ICV-ERT and HSCT in a patient with neuronopathic MPS II [21]. The patient was diagnosed at 2 months of age with a suspected neuronopathic phenotype, based on family history and genetic variant patterns.

Another recently available ERT agent, pabinafusp alfa (IZCARGO^®^), which consists of human I2S fused to a BBB-crossing anti-transferrin receptor antibody, is also effective against CNS symptoms [15,16], even in patients with severe genetic mutations. Early treatment tends to improve DA, similar to ICV-ERT. Although the long-term clinical outcomes and the effects in asymptomatic patients diagnosed via NBS remain unclear, the decrease in CSF HS concentrations confirms that the agent crosses the BBB; however, its efficacy in improving neurological symptoms is currently less pronounced than that of ICV-ERT with idursulfase beta (Hunterase^®^). Furthermore, as only approximately 1% of intravenously administered pabinafusp alfa is reported to reach the brain [22], a dosage of 2 mg/kg may be insufficient to achieve adequate therapeutic efficacy in certain patients. Although pabinafusp alfa is less invasive than ICV-ERT, weekly administration is required. Thus, we combined HSCT with ICV-ERT to leverage the practical advantage of monthly hospital visits. Admittedly, ICV-ERT carries a perceived risk of serious complications such as bacterial meningitis; however, in a previous 5-year clinical trial involving 6 patients with MPS II, no cases of bacterial meningitis were observed despite approximately 1000 scheduled doses being administered [14]. This clinical evidence indicates that ICV-ERT via an implanted reservoir does not pose an inherent risk when performed with appropriate caution.

Another major factor in this decision was the recent improvement in the treatment outcomes of HSCT for inborn errors of metabolism (IEM) [23], which has a major influence on the success of HSCT for IEM because these children are immunocompetent. Therefore, myeloablative conditioning was traditionally performed, which increases the risk of transplant-related morbidity and mortality. Since the 2000s, the introduction of reduced-intensity conditioning instead of myeloablative conditioning has decreased treatment-related mortality without increasing engraftment failure [24]. CBT is preferred over unrelated donor bone marrow transplantation due to its many advantages, including less stringent HLA matching requirements, lower risk of severe GVHD, and lack of risk for the donor [25]. Although the small number of cells obtained is one of the disadvantages of CBT, this is less of an issue when treating small infants; in this regard, early diagnosis through NBS increases the likelihood of successful engraftment. However, HSCT can cause life-threatening complications. For example, one of our patients experienced SOS but recovered without sequelae, which is attributed to the scoring assessment using abdominal ultrasound-based scoring for SOS [26] and the administration of a highly effective drug such as defibrotide [27]. Advances in the treatment of transplantation-related complications provide major benefits. HSCT allows donor-derived enzyme-producing cells to migrate to the brain, thereby enabling permanent ERT [28]. However, whether HSCT alone is sufficient to prevent CNS manifestations in asymptomatic patients with MPS II identified early through NBS remains uncertain. The study results suggest that, in our cases, the concentration of HS in the CSF increased after the interruption of ICV-ERT during the acute therapeutic stage of HSCT. Recent evidence has demonstrated that CSF HS is a biomarker for neuronopathic MPS [29]. Although no CNS symptoms were observed, the long-term effects of HSCT alone on CNS disease control appear to be insufficient. This study was limited by the small number of cases and unresolved issues, such as how to manage antibody development. Furthermore, if ex vivo gene therapy using lentiviruses becomes practical, ICV-ERT may become unnecessary [30]. However, at present, we propose that combining ICV-ERT and CBT is optimal for treating neuronopathic MPS II.

The prevalence of MPS II is high in East Asia [31]. NBS for MPS II began in Taiwan in 2015 and in Illinois and Missouri in 2018. Following a 2022 recommendation from the US Recommended Uniform Screening Panel, MPS II screening has expanded to other states in the US [32]. Early diagnosis through NBS clearly contributed to the favorable outcomes observed in our patients. In Taiwan, over the decade leading up to 2025, a total of 14 patients with MPS II were diagnosed during asymptomatic infancy, followed by the initiation of disease-specific early interventions including ERT and HSCT [33]. Our next challenge is to prepare for the implementation of standardized NBS procedures for all newborns in Japan, as cases may remain undiagnosed under the current system.

In conclusion, NBS is likely to be particularly valuable in Japan, where there is a high prevalence of MPS II and CNS treatments are available. Combination therapy with ICV-ERT followed by CBT may improve the neurological prognosis of patients diagnosed with MPS II at an early stage of life.

## Figures and Tables

**Table 1:** The profiles of patients.

Patient		1	2	3	Patient 3 Is an Older Brother of Patient 2
Results of NBS	I2S (4-MU method)	0.000	0.000	N/A	Cut off < 4 pmol/h/disk
	I2S (MS/MS method)	0.379	0.231	N/A	Cut off < 2.9 μM/h
Initial diagnostic examinations	Urinary GAGs	158	99	39	RI: 24.7 ± 10.7 mg/mmol cre
	Urinary uronic acids	438	N/A	137	RI: 43.4 ± 12.9 mg/g cre
Confirmatory examinations	I2S enzyme activity	<0.9	<0.9	<0.9	
	*IDS* gene variant	c.685 C > T	c.1180 + 1 G > T	c.1180 + 1 G > T	
Symptoms	Mongolian spots	-	+	+	
	Facial features	-	Prominent forehead	Prominent forehead	
	Enlarged abdomen	-	-	-	
	Umbilical hernia	-	-	-	
	Joint stiffness	-	-	-	
	Developmental delay	-	-	Mild	
ERT	Age at starting intravenous ERT	3 mo	6 mo	2 y and 5 mo	
	Complications of intravenous ERT	-	-	-	
	Age at starting ICV-ERT	5 mo	11 mo	2 y and 7 mo	
	Complications of ICV-ERT	Fever, nausea	Fever, nausea	Fever, nausea	

NBS, newborn screening; I2S, iduronate-2-sulfatase; 4-MU, 4-methylumbelliferone; MS/MS, tandem mass spectrometry; N/A, not available; GAGs, glycosaminoglycans; RI, reference interval; ERT, enzyme replacement therapy; ICV, intracerebroventricular.

**Table 2:** The clinical course of cord blood transplantation.

Patient	Age	HLA Compatibility	Numbers of CD34-Positive Cells (/kg)	Neutrophil Engraftment Period (Days)	Period Until Platelet Counts > 50,000 (Days)	Grade of Acute GVHD	Grade of Chronic GVHD	Acute AE	Chronic AE	I2S Enzyme Activity	The Interval of Re-Administration of ICV-ERT (Days)
1	2 y and 6 mo	8/8	2.0 × 10^5^	22	62	II	Mild	SOS	Hypothyroidism	86	68
2	1 y and 8 mo	6/8	2.5 × 10^5^	15	56	I	Mild	-	-	132	76
3	3 y and 3 mo	8/8	4.4 × 10^5^	16	32	I	Mild	-	-	57.9	55

GVHD, graft-versus-host disease; AE, adverse event; ICV, intracerebroventricular; ERT, enzyme replacement therapy; HLA, Human Leukocyte Antigen; y, year; mo, month; I2S, iduronate-2-sulfatase; SOS, sinusoidal obstruction syndrome.

## Data Availability

The original contributions of this study are included in the manuscript. Further inquiries can be directed to the corresponding author.

## Abbreviations

The following abbreviations are used in this manuscript:

MPS II	Mucopolysaccharidosis type II
NBS	Newborn screening
ICV	Intracerebroventricular
ERT	Enzyme replacement therapy
ICV-ERT	Intracerebroventricular enzyme replacement therapy
CBT	Cord blood transplantation
HS	Heparan sulfate
LSDs	Lysosomal storage disorder
I2S	Iduronate-2-sulfatase
GAGs	Glycosaminoglycans
CNS	Central nervous system
HSCT	Hematopoietic stem cell transplantation
BBB	Blood–brain barrier
4-MU	4-methylumbelliferone
MS/MS	Tandem mass spectrometry
GVHD	Graft-versus-host disease
CSF	Cerebrospinal fluid
DA	Developmental age
DQ	Developmental quotient
KSPD	Kyoto Scale of Psychological Development
SOS	Sinusoidal obstruction syndrome
IEM	Inborn errors of metabolism
